# Countries’ response to WHO’s travel recommendations during the 2013–2016 Ebola outbreak

**DOI:** 10.2471/BLT.16.171579

**Published:** 2016-10-18

**Authors:** Wendy Rhymer, Rick Speare

**Affiliations:** a College of Public Health, Medical and Veterinary Sciences, James Cook University, Townsville, Queensland 4811, Australia.; b Deceased, formerly College of Public Health, Medical and Veterinary Sciences, James Cook University.

## Abstract

**Objective:**

To determine how, during the 2013–2016 Ebola outbreak in western Africa, States Parties to the World Health Organization’s (WHO) 2005 International Health Regulations (IHR) followed the IHR’s international travel recommendations.

**Methods:**

In 2015, we used the Google search engine to investigate the 196 States Parties to the 2005 IHR. Information detailing Ebola-related travel regulations or restrictions of each State Party was sourced first from official government websites and then from travel and news websites. When limited, conflicting or no relevant information was found on a government website, an email inquiry was sent to a corresponding embassy in an Anglophone country.

**Findings:**

We collected relevant and non-conflicting data for each of 187 States Parties. Of these, 43 (23.0%) prohibited the entry of foreigners who had recently visited a country with widespread Ebola transmission and another 15 (8.0%) imposed other substantial restrictions on such travellers: the requirement to produce a medical certificate documenting no infection with Ebola (*n* = 8), mandatory quarantine (*n* = 6) or other restrictions (*n* = 1).

**Conclusion:**

In responding to the 2013–2016 Ebola outbreak, countries had variable levels of adoption of the 2005 IHR’s international travel recommendations. We identified 58 (31.0%) States Parties that exceeded or disregarded the recommendations. There is a need for more research to understand and minimize deviations from such recommendations.

## Résumé

**Objectif:**

Déterminer comment, lors de la flambée de maladie à virus Ebola qui a sévi de 2013 à 2016 en Afrique de l'Ouest, les États parties au Règlement sanitaire international (RSI) de 2005 de l'Organisation mondiale de la Santé (OMS) ont suivi les recommandations du RSI concernant les voyages internationaux.

**Méthodes:**

En 2015, nous avons utilisé le moteur de recherche Google pour mener une enquête sur les 196 États parties au RSI de 2005. Nous avons d'abord recherché des informations sur la règlementation ou les restrictions relatives aux voyages appliquées par chaque État partie pour faire face à la flambée de maladie à virus Ebola sur les sites Internet officiels des gouvernements, puis sur des sites Internet de voyage et d'information. Lorsque les informations disponibles sur le site Internet d'un gouvernement étaient insuffisantes, contradictoires ou non pertinentes, une demande a été envoyée par e-mail à l'ambassade correspondante dans un pays anglophone.

**Résultats:**

Nous avons recueilli des données pertinentes et non contradictoires sur 187 États parties. Sur ces 187 États parties, 43 (23,0%) ont interdit l'entrée sur leur territoire aux étrangers ayant récemment séjourné dans un pays où la transmission de la maladie à virus Ebola prenait une ampleur considérable et 15 (8,0%) ont imposé d'autres restrictions importantes à ce type de voyageurs: présentation obligatoire d'un certificat médical attestant que le voyageur n’est pas infecté par le virus Ebola (*n* = 8), quarantaine obligatoire (*n* = 6) ou autres restrictions (*n* = 1).

**Conclusion:**

Face à la flambée de maladie à virus Ebola qui a sévi entre 2013 et 2016, les pays ont adopté à différents niveaux les recommandations du RSI de 2005 concernant les voyages internationaux. Nous avons repéré 58 (31,0%) États parties ayant dépassé ou ignoré les recommandations. Il est nécessaire de mener davantage de recherches pour comprendre et réduire les entorses faites à ces recommandations.

## Resumen

**Objetivo:**

Determinar cómo los Estados Partes del Reglamento Sanitario Internacional (RSI) de la Organización Mundial de la Salud (OMS) siguieron las recomendaciones sobre viajes internacionales del RSI de 2005 durante el brote de ebola en el oeste africano entre 2013 y 2016.

**Métodos:**

En 2015, se utilizó el motor de búsqueda de Google para investigar los 196 Estados Partes del RSI de 2005. La información que detallaba los reglamentos sobre viajes relacionados con el ebola o las restricciones de cada Estado Parte se obtuvo primero de los sitios web gubernamentales oficiales y, posteriormente, de sitios web sobre viajes y noticias. Cuando la información era escasa, contradictoria o no se encontraba en el sitio web gubernamental, se enviaba un correo electrónico de solicitud a la embajada correspondiente de un país angloparlante.

**Resultados:**

Se recopilaron datos importantes y coherentes de 187 Estados Partes. Entre ellos, 43 (23,0%) prohibieron la entrada de extranjeros que habían visitado recientemente un país con un nivel generalizado de contagio del ebola y otros 15 (8,0%) impusieron otras restricciones importantes para dichos viajeros: el requisito de obtener un certificado médico que documentara que el individuo no estaba infectado con el virus (*n* = 8), cuarentena obligatoria (*n* = 6) u otras restricciones (*n* = 1).

**Conclusión:**

Como respuesta al brote de ebola entre 2013 y 2016, los países contaban con distintos niveles de adopción de las recomendaciones sobre viajes internacionales del RSI de 2005. Se identificaron 58 (31,0%) Estados Partes que superaron o incumplieron las recomendaciones. Existe la necesidad de una búsqueda más exhaustiva para comprender y minimizar los incumplimientos de dichas recomendaciones.

## ملخص

**الغرض:**

الوقوف على مدى التزام الدول الأطراف في اتفاقية اللوائح الصحية الدولية الصادرة عن منظمة الصحة العالمية لعام 2005 باتباع توصيات السفر الدولية المنصوص عليها في تلك اللوائح وذلك خلال تفشي فيروس الإيبولا في الفترة من 2013-2016 بغرب أفريقيا.

**الطريقة:**

في عام 2015، استخدمنا محرك البحث "جوجل" لتحري الدول الأطراف في اتفاقية اللوائح الصحية الدولية الصادرة عام 2005، والبالغ عددها 196 دولة. وتم في البداية استخلاص المعلومات التي تشرح بالتفصيل لوائح أو قيود السفر المتعلقة بفيروس الإيبولا والمفروضة في كل دولة من الدول الأطراف من المواقع الإلكترونية الحكومية الرسمية ثم المواقع الإلكترونية للسفر والأخبار. وعند العثور على معلومات محدودة أو متضاربة أو أي غير متصلة على الموقع الإلكتروني للحكومة، كان يتم إرسال رسالة استفهامية بالبريد الإلكتروني إلى السفارة المناظرة في إحدى البلاد الناطقة بالإنجليزية.

**النتائج:**

لقد جمعنا بيانات ذات صلة لا يشوبها التضارب لكل من 187 دولة من الدول الأطراف. ومن بين تلك الدول كانت 43 (بواقع 23.0‏٪) تحظر دخول الأجانب الذين زاروا مؤخرًا بلدًا يتفشى بها فيروس الإيبولا على نطاق واسع، فيما كانت 15 دولة أخرى (بواقع 8.0‏٪) تفرض قيودًا كبيرة على هؤلاء المسافرين، إذ كانت تشترط تقديم شهادة طبية شهادة طبية تثبت عدم الإصابة بعدوى الإيبولا ( *بلغ عددها * = 8)، أو الحجر الصحي الإلزامي ( *بلغ عددها * = 6) أو غيرها من القيود ( *بلغ عددها * = 1).

**الاستنتاج:**

في إطار الاستجابة لتفشي فيروس الإيبولا بين عامي 2013-2016، فقد أظهرت البلدان مستويات مختلفة من اعتماد توصيات السفر الصحية الدولية الصادرة عام 2005. وقد حددنا 58 دولة (بواقع 31.0‏٪) من الدول الأطراف التي تجاوزت التوصيات أو تجاهلتها. وهناك حاجة لإجراء المزيد من الأبحاث لفهم الانحرافات عن تلك التوصيات والحد منها.

## 摘要

**目的:**

旨在确定 2013—2016 年西非埃博拉病毒爆发期间，世界卫生组织 (WHO) 2005 年《国际卫生条例》 (IHR) 缔约国对《IHR 国际旅行指南》的采用情况。

**方法:**

2015 年，我们使用谷歌 (Google) 搜索引擎调查了 196 个缔约国对 2005 年 IHR 指南的采用情况。 我们首先从各缔约国政府网站上获取了各国与埃博拉病毒相关的旅行规定或限制详情，然后从旅行和新闻网站获取了相关信息。 如有限制，冲突或在政府网站上未找到相关信息，可发电子邮件至讲英语国家的相应大使馆。

**结果:**

我们收集了 187 个缔约国中各个国家的相关和非冲突数据。 其中，43 (23.0%) 个国家禁止近期去过埃博拉病毒大面积传播国家的外籍人士入境，另有 15 (8.0%) 个国家对此类游客采取了其他实质性限制措施： 需要提供未感染埃博拉病毒的医学证明 (*n* = 8)，必须进行隔离检疫 (*n* = 6) 或其他限制措施 (*n* = 1)。

**结论:**

为应对 2013–2016 年的埃博拉病毒爆发，各国都不同程度地采用了 2005 年《IHR 国际旅行指南》。 我们发现有 58 (31.0%) 个缔约国过度使用或忽视该指南。 我们还需要做更多的研究以理解此类指南，同时在最大程度上减少对指南的理解偏差。

## Резюме

**Цель:**

Определить, как во время вспышки лихорадки Эбола в Западной Африке в 2013–2016 годах страны-участники соблюдали рекомендации по международным поездкам в рамках Международных медико-санитарных правил (ММСП) от 2005 года Всемирной организации здравоохранения (ВОЗ).

**Методы:**

В 2015 году с помощью поисковой системы Google было проведено исследование 196 стран-участников соблюдения ММСП 2005 года. Из каждой страны-участника была получена информация о постановлениях или ограничениях поездок в связи с лихорадкой Эбола, в первую очередь с официальных правительственных, а затем с туристических и новостных веб-сайтов. В случае если информация на правительственном сайте была неполной, противоречивой или не освещала исследуемую тему, в соответствующее посольство в англоязычной стране направлялся запрос по электронной почте.

**Результаты:**

Релевантные и непротиворечащие данные были получены для каждой из 187 стран-участников соблюдения ММСП. В 43 из них (23,0%) был запрещен въезд иностранных граждан, незадолго до этого посещавших страну, на территории которой были широко распространены случаи заражения вирусом Эбола; в других 15 (8,0%) были введены иные существенные ограничения для совершивших опасную поездку граждан, а именно требование предоставить медицинскую справку об отсутствии заболевания Эбола (*n* = 8), обязательный карантин (*n* = 6) или другие ограничения (*n* = 1).

**Вывод:**

В ответ на вспышку эпидемии Эбола в 2013–2016 годах страны в разной степени внедрили рекомендации в отношении международных поездок, предложенные в рамках ММСП 2005 года. Авторы установили, что 58 стран-участников (31,0%) вышли за рамки данных рекомендаций или вовсе проигнорировали их. Требуются дополнительные исследования, чтобы понять и минимизировать данные отклонения. исследования, чтобы понять и минимизировать данные отклонения.

## Introduction

Ebola virus disease – previously known as Ebola haemorrhagic fever – was first identified in 1976 in Zaire – the country now known as the Democratic Republic of Congo.^1^ The disease is severe and often fatal. The causative virus is initially transmitted from wild animals to humans but is then spread through the human population by direct contact with infected, symptomatic individuals or their blood, body fluids or secretions.^2^ Infected individuals are only infectious when symptomatic and become symptomatic two to 21 days after infection.^2^

The 2013–2016 Ebola outbreak in western Africa was the longest and largest on record.^1^ The index case was identified as a boy, aged two years, who became ill, on 28 December 2013, in the remote Guinean village of Meliandou.^3^ The virus spread to neighbouring countries via travellers crossing land borders.^4^ By 20 January 2016, the World Health Organization (WHO) had reported 28 602 confirmed, probable or suspected cases of Ebola virus disease, including 11 301 fatal cases, in the outbreak.^5^ The end of an Ebola outbreak in a country is declared 42 days after the blood of the country’s last confirmed case has twice tested negative for the virus.^6^ On 14 January 2016, WHO declared Liberia to be free of transmission and, in consequence, the outbreak in western Africa to be ended.^7^ However, one day later, WHO confirmed the presence of a new case of Ebola in Sierra Leone – a country that had been declared Ebola-free on 7 November 2015.^8^ Guinea had been declared free of Ebola transmission on 29 December 2015.^9^ During the outbreak, small numbers of cases were reported in Mali, Nigeria and Senegal and also beyond western Africa – e.g. in Italy, Spain, the United Kingdom of Great Britain and Northern Ireland and the United States of America (USA).^10^

The pattern of spread in western Africa suggested that international travel was key to the widespread transmission of Ebola virus in the outbreak. Nigeria’s index case flew from Liberia to the Nigerian city of Lagos after caring for a sibling who subsequently died from Ebola^11^ and, from this case, another 19 individuals in Nigeria became infected.^12^ Similarly, the index case in Senegal had direct contact with an Ebola patient in Guinea before travelling, by road, to Dakar – the capital city of Senegal.^13^ The index case in the United States presented, in September 2014, after having flown from Liberia – although the level of contact this case had with any Ebola cases in western Africa remains unclear.^14^^,^^15^ Two nurses who had cared for this case, in the American city of Dallas, developed Ebola virus disease.^15^ In October 2014, a health-care worker in Spain tested positive for the disease after caring for a repatriated medical missionary who had previously worked in a hospital in Sierra Leone.^16^ This Spanish index case was the first known case of secondary transmission of Ebola virus outside Africa.^16^ Also in October 2014, Mali identified their index case to be a young resident of Guinea who had travelled, by road, to Kayes, Mali, after family members had died of Ebola virus disease in Guinea.^17^ The index cases in Italy and the United Kingdom were both health-care workers who had returned from working in Ebola treatment centres in Sierra Leone.^18^^,^^19^

In the 2005 International Health Regulations (IHR), which were implemented on 15 June 2007, 196 States Parties to the IHR agreed that early detection of – and response to – a disease can decrease the rate of transmission and lessen the negative impacts on health and society.^20^^,^^21^ Each of the States Parties to the IHR agreed to maintain disease surveillance, share public health information of international significance and support other countries.^20^ During public health emergencies, the IHR help to guide the WHO Director-General’s recommendations about international trade and travel.^20^

The so-called public health emergency of international concern is an innovation of the 2005 IHR regarding global emergency responses to certain public health dangers.^20^ Such an emergency is defined as “an extraordinary event which is determined to constitute a public health risk to other States through the international spread of disease and to potentially require a coordinated international response”.^20^ When an emergency of this type is anticipated, WHO’s Director-General convenes a group of experts – known as the IHR Emergency Committee – to advise on the determination of a public health emergency of international concern and temporary recommendations on public health measures.^21^ To prevent disease spread and minimize the impact of the emergency on international travel and trade, each State Party is expected to follow this advice and implement appropriate disease surveillance and control at national level.^21^

On 8 August 2014, WHO’s Director-General declared the Ebola outbreak then occurring in western Africa to be a public health emergency of international concern.^4^ This was only the third such emergency to be declared; the fourth, for the spread of Zika virus, would not be declared until February 2016.^22^^,^^23^ Countries with Ebola transmission were advised to begin exit screening, at all international airports, land crossings and seaports, for febrile illness of unknown origin, that was consistent with Ebola virus disease.^24^ The advice included travel restrictions for all confirmed, probable, suspected or contact cases of Ebola virus disease until either Ebola virus disease could be ruled out or recovery verified – unless the travel formed part of a medical evacuation plan.^24^ General bans on international travel were not advised and, although such screening was not recommended, countries that implemented entry screening were asked to share any lessons learnt.^25^

As soon as the Ebola-related public health emergency was declared and the recommendations publicized, most countries began to respond and make implementation decisions. National responses to the declaration varied from complete adoption to complete disregard – including implementation of general travel bans. There is little information available on how countries respond in general to WHO’s declarations of emergencies and, as yet, there has been no comprehensive study on national responses to such declarations.

In the present study, our aim was to investigate the compliance of the States Parties to the 2005 IHR with the international travel recommendations made by WHO when declaring the Ebola-related public health emergency. We were particularly interested in how such States Parties intended to handle foreign travellers who had recently visited countries with widespread Ebola transmission.

## Methods

Between 9 March 2015 and 8 April 2015, we used the Google search engine (Google, Mountain View, USA) to search websites for relevant information on each of the 196 State Parties to the 2005 IHR.^26^ The initial search terms were the name of a State Party plus “Ebola” and at least one of the following: “WHO”, “World Health Organization”, “IHR” and “International Health Regulations”. For each State Party, a minimum of 20 and a maximum of 100 hits were visited. If an official website for the State Party – e.g. a website with a uniform resource locator that included the domain name gov – could not be found, the search terms were expanded to cover (i) the name of a State Party plus both “Ebola” and “travel” and at least one of the following: “regulations”, “restrictions” and “recommendations” or (ii) the name of a State Party plus both “Ebola” and either “Ministry” or “Department” and at least one of the following: “of Health”, “of foreign affairs”, “of health and welfare” and “of immigration”.

The search was stopped when an official government site was found that gave details on Ebola-related travel regulations or restrictions. If an official government site was not identified, then travel and news websites were checked. Google Translate (Google, Mountain View, USA) was used for the translation of web pages into English as well as the translation of the search terms from English into a country’s official language.

When limited, conflicting or no relevant information was found on a State Party’s official government website, we sent an email to an embassy of the State Party in an Anglophone country – i.e. Australia, Canada, the United Kingdom or the United States – inquiring about any travel regulations or restrictions for travellers who had been working in Ebola-affected countries. 

If no useful information on a State Party was gathered after web searches and emails to embassies or if conflicting reports could not be clarified, the search for information on that State Party was halted.

We used Excel (Microsoft, Redmond, USA) databases to store the information we collected. States Parties were categorized according to WHO region and income grouping.^27^ As well as details of the Ebola-related measures enforced by each State Party, we recorded the date information was sourced, the date the information was posted on the website, the website address and any information from emails that assisted with identifying the regulations. We separated measures into those that permitted unconditional entry of all foreign travellers who had recently visited countries with widespread Ebola transmission and those in which entry was conditional. We collected data on whether and, if so, how screening was done on entry, whether a medical certificate was required, whether information on Ebola was distributed and if any monitoring or quarantine was implemented. Case studies, to illustrate each main category of response, were also assembled.

James Cook University Human Research Ethics Committee, Townsville, Australia, approved the study protocol, via approval H6043.

## Results

We collected relevant non-conflicting data on 187 (95.4%) of the 196 States Parties to the 2005 IHR. We were unable to collect such data for Guinea, Guinea-Bissau, Libya, Niger, Palau, Somalia, Timor-Leste, Vanuatu or Yemen. For 126 (67.4%) States Parties, the main source of the data we analysed was an official government website. A travel website was the main source for 26 (13.9%) States Parties, while a news website and email correspondence with an embassy or health department were the main source for 22 (11.8%) and 13 (7.0%) States Parties, respectively.

Overall, we found that 58 (31.0%) of the States Parties in our analysis had exceeded or disregarded the 2005 IHR’s international travel recommendations. Entry of foreigners who had departed from a country with widespread transmission of Ebola was prohibited in 43 (23.0%) (Table 1) and another 15 (8.0%) of the States Parties had applied exclusions or substantial restrictions to such travellers. Eight had the requirement to produce a medical certificate documenting no infection with Ebola, six had mandatory quarantine and one allowed the entry of foreigners who had been working in Ebola-affected countries while denying the entry of citizens from such countries (Table 1).

**Table 1:** Countries banning entry of travellers from countries with widespread Ebola virus transmission or allowing entry with substantial restrictions, March–April 2015

Country	Entry restrictions	Data source
Afghanistan	Exclude if no certificate	Government website
Algeria	Exclude if no certificate	Travel website
Antigua and Barbuda	No entry	Government website
Australia	No entry	Government website
Bahrain	No entry	Government website
Belize	No entry	Government website
Botswana	No entry	Government website
Cabo Verde	No entry	Travel website
Cameroon	No entry	Travel website
Canada	No entry	Government website
Central African Republic	No entry	Travel website
Chad	No entry	Travel website
Colombia	No entry	Government website
Democratic People's Republic of Korea	Entry but mandatory quarantine	Travel website
Dominica	No entry	Government website
Dominican Republic	No entry	Government website
Equatorial Guinea	No entry	Travel website
Gabon	No entry	Travel website
Gambia	No entry	Travel website
Guyana	No entry	News website
Haiti	No entry	Government website
Indonesia	Exclude if no certificate	Email correspondence with embassy
Iraq	Exclude if no certificate	Travel website
Jamaica	No entry	Government website
Kazakhstan	Exclude if citizen of Ebola-affected country	Email correspondence with embassy
Kenya	No entry	Travel website
Kiribati	Entry but mandatory quarantine	Email correspondence with health department
Kuwait	No entry	News website
Maldives	No entry	Government website
Mauritania	No entry	Travel website
Mauritius	No entry	Government website
Micronesia (Federated States of)	No entry	Government website
Mongolia	No entry	Travel website
Namibia	No entry	Travel website
Nauru	No entry	Government website
Nepal	Exclude if no certificate	Email correspondence with embassy
Nicaragua	Entry but mandatory quarantine	Travel website
Panama	No entry	Government website
Peru	Exclude if no certificate	Email correspondence with embassy
Philippines	Entry but mandatory quarantine	Travel website
Qatar	No entry	News website
Republic of Korea	Entry but mandatory quarantine	Government website
Romania	No entry	Government website
Rwanda	No entry	Government website
Saint Kitts and Nevis	No entry	Government website
Saint Lucia	No entry	Government website
Saint Vincent and the Grenadines	No entry	Government website
Sao Tome and Principe	No entry	Travel website
Saudi Arabia	No entry	Government website
Serbia	Entry but mandatory quarantine	Government website
Seychelles	No entry	Government website
South Africa	No entry	Government website
South Sudan	No entry	Government website
Suriname	No entry	Travel website
Trinidad and Tobago	No entry	Email correspondence with embassy
Turkmenistan	Exclude if no certificate	Email correspondence with embassy
Tuvalu	Exclude if no certificate	Email correspondence with embassy
Zambia	No entry	Government website

Details on monitoring after entry of foreigners from countries with widespread transmission were provided by 107 (74.3%) of the 144 States Parties allowing entry (Table 2).

**Table 2:** Ebola-related interventions on the borders of 144 countries allowing entry of travellers from countries with widespread Ebola transmission, March–April 2015

Intervention	No. (%) of countries
**Assessment of risk level**	4 (2.8)
**EVD information provided**	15 (10.4)
**Investigation of travel health history**	22 (15.3)
**Medical examination**	5 (3.5)
**Monitoring**	
Only by travellers	12 (8.3)
Only by health department	25 (17.4)
By both travellers and health department	1 (0.7)
**Quarantine**	6 (4.2)
**Recording of body temperature**	24 (16.7)
**Registration**	3 (2.1)
**Screening**	85 (59.0)

EVD: Ebola virus disease.

Our data were incomplete, in terms of the Member States covered, for all but two WHO regions (Table 3). Although every WHO region had at least one Member State that prohibited entry, such prohibition was most common among the Member States of the African Region and the Region of the Americas (Table 3).

**Table 3:** Prohibition of the entry of foreign travellers from Ebola-affected countries, March–April 2015

Country classification	No. of countries	No. (%)	
		Countries with data available	Countries prohibiting entry
**WHO region**			
African Region	47	44 (93.6)	18 (38.3)
Region of the Americas	35	35 (100.0)	15 (42.9)
South-East Asia Region	11	10 (90.9)	1 (9.1)
European Region	53^a^	53 (100.0)	1 (1.9)
Eastern Mediterranean Region	21	18 (85.7)	4 (19.0)
Western Pacific Region	27	25 (92.6)	4 (14.8)
All	194	185 (95.4)	43 (22.2)
**Country income group**			
High	55	55 (100.0)	10 (18.2)
Upper middle	36	32 (88.9)	7 (19.4)
Lower middle	57	55 (96.5)	18 (31.6)
Low	46	43 (93.5)	8 (17.4)

WHO: World Health Organization.

^a^ Excluding two of the States Parties to the 2005 International Health Regulations – i.e. the Holy See and Lichtenstein – as they only have observer status for the European Region.

Our analysis covered every high-income country and the majority of countries in each of the lower income groupings (Table 3). Within each income group there was at least one country that prohibited entry of foreigners who had departed from a country with widespread transmission of Ebola (Table 3).

### Case studies

The following case studies provide examples of States Parties to the 2005 IHR that had fully adopted WHO’s recommendations on the Ebola-related public health emergency, had prohibition of entry that disregarded the recommendations or appeared to have other restrictions that exceeded WHO’s recommended response.

#### Full adoption of recommendations

On 8 August 2014, in a joint statement by the Minister of Foreign Affairs and International Development and the Minister of Social Affairs and Health, France welcomed the decisions and recommendations made, by the IHR Emergency Committee and WHO, on Ebola-related responses.^28^ France agreed to meet all WHO recommendations when implementing Ebola-related preventative measures, treatment preparations and public health information campaigns.^28^ Temperature screening was started for passengers on direct flights or ships from Ebola-affected countries, and, on arrival, such passengers were provided with information leaflets on Ebola, in case they became ill in the following 21 days.^29^

#### Prohibition of entry

On 28 October 2014, the Australian Department of Immigration announced the temporary suspension of all visa application assessments for citizens of Ebola-affected countries^30^ and the possible cancelation of the visas of individuals who were currently outside Australia and had been in an Ebola-affected country within the previous 21 days.^31^ These restrictions were subsequently extended to cover all individuals who were not Australian citizens or permanent residents – including foreigners who had recently visited Ebola-affected countries.^32^ If individuals were able to prove that they had not been in an Ebola-affected country within the previous 21 days and did not plan to travel to such a country before entering Australia, they were allowed to reapply or seek revocation of the decision to cancel their visa, pending their examination by a panel physician.^32^^,^^33^ Australia stated that this was not a travel ban and that the new regulations would not impede the assistance that Australia could give to Ebola-affected countries.^31^

#### Additional restrictions

From 1 February 2015, Afghanistan required that all foreign passport holders have a visa properly prepared, or in their possession, before their arrival in the country.^34^ To obtain a visa, each applicant was to have a recent health certificate, from a doctor, that proved that the applicant was free from Ebola.^34^ Without this certificate of health, a visa could be denied.^34^

## Discussion

Although the Ebola outbreak that formed the basis of our study was the third public health emergency of international concern to be declared, our study appears to be the first attempt to assess the adherence of countries’ responses to the 2005 IHR. Of the States Parties with accessible information relevant to our study, 23.0% had imposed a ban on the entry of foreigners travelling from countries with widespread transmission of Ebola. This response conflicts with the 2005 IHR, which state that there is to be no such general ban on international travel.^20^ Of the States Parties that allowed entry of such foreigners, 8.0% had other substantial restrictions or entry exclusions, none of which followed the detailed recommendations of the IHR Emergency Committee or WHO on the Ebola emergency. However, the 2005 IHR do not preclude countries from implementing health measures that “achieve the same or greater level of health protection than WHO recommendations” as long as those “measures shall not be more restrictive of international traffic and not more invasive or intrusive to persons than reasonably available alternatives that would achieve the appropriate level of health protection.”^20^

The consequences of countries creating their own policies and regulations, irrespective of any international recommendations, include the possibility of practices being implemented that are not based on scientific evidence. For example, Afghanistan required a medical certificate stating that the person was free from Ebola.^34^ However, the incubation period of Ebola virus disease is up to 21 days and diagnostic tests based on the polymerase chain reaction may give a negative result, for an infected individual, until the third day of symptoms.^35^ In consequence, a medical certificate based on the result of such a diagnostic test does not guarantee that the person has not been infected or that the person will not develop Ebola virus disease up to three weeks later.^35^ The result of such certification may be a false sense of security in the traveller, the border officials at the point of entry and the community at large.

Another potential consequence of countries choosing to sidestep the intentions of the 2005 IHR is the introduction of discriminatory policies. At one stage of the Ebola outbreak, Australia was restricting the entry of everyone who was not an Australian citizen or an Australian permanent resident.^30^^–^^33^ Although the Iraqi government required a health-clearance certificate for almost all travellers entering Iraq who had visited an Ebola-affected country, holders of diplomatic passports were exempt from providing a certificate.^36^ Such exemptions for potentially at-risk individuals conflict with the IHR, which encourage countries to work together to prevent and respond to global health emergencies.^35^

The lack of any clear negative consequences for States Parties that decide to disregard the recommendations within the 2005 IHR is a weakness of the regulations. WHO states that peer pressure and public knowledge are the best incentives for adoption of the recommendations, since the “consequences of non-compliance include a tarnished international image, increased morbidity/mortality of affected populations, unilateral travel and trade restrictions, economic and social disruption and public outrage”.^37^ Given the regulations and restrictions imposed by States Parties during the 2013–2016 Ebola outbreak, many countries appear undeterred by the consequences of their non-adoption of the recommendations.

In general, access for travellers to information regarding countries’ Ebola-related travel regulations appeared to be inadequate. While information regarding these regulations was available for almost all of the States Parties to the 2005 IHR, a quarter of that information came from unofficial sites, such as news and travel sites, and was sometimes incomplete. Travellers need accurate information on a country’s entry requirements before they arrive at that country’s border.

Our study had several limitations. For nine States Parties we were unable to find relevant information or it was incomplete and of poor quality or reliability. Our approach to data did not take into account the communication of travel regulations and whether or not, in any State Party, the national IHR focal point was consulted during Ebola-related decision-making. We did not attempt to assess the level and consistency of the implementation of the adopted recommendations at international entry points. Furthermore, the information we analysed was collected about seven months after the Ebola-related public health emergency was announced. At that time, with the incidence of Ebola disease in decline, some countries had loosened their restrictions on – and recommendations for – travellers. In addition, our reliance on Google Translate to access information that was not in English may have led to relevant information being missed or misunderstood. In future related studies, we would recommend contact with IHR focal points and/or local agencies for the control of communicable diseases.

In conclusion, our study shows that countries had variable levels of adoption of the WHO international travel recommendations made in response to the 2013–2016 Ebola outbreak. We identified about a third of States Parties that exceeded or disregarded the recommendations. There is a need for more research to understand and minimize deviations from such recommendations.
